# Small Bowel Does Not Belong in the Pericardium: Case of a Traumatic Intrapericardial Diaphragmatic Hernia

**DOI:** 10.7759/cureus.32966

**Published:** 2022-12-26

**Authors:** Vitaliy Dushnov, Meagan McNicholas, Mingda Su, Papus Keita, Henry Moore

**Affiliations:** 1 Surgery, Carle Foundation Hospital, Urbana, USA; 2 Cardiothoracic Surgery, Carle Illinois College of Medicine, Urbana, USA; 3 Surgery, Carle Illinois College of Medicine, Urbana, USA

**Keywords:** laparoscopic hernia repair, laparoscopic surgery, diaphragmatic hernias, trauma, intrapericardial hernia

## Abstract

Intrapericardial diaphragmatic hernia (IPDH) is rare and most often a sequela of blunt thoracic trauma. The trans-abdominal or thoracic repair approaches are based on the acuteness of presentation and the expectation of encountering intrapericardial adhesions. We present an acute IPDH in an 80-year-old female patient managed with a laparoscopic trans-abdominal repair. Misdiagnosis and complications from the delayed presentation can be avoided with careful attention to the initial exam, imaging, and early operative repair if the patient is a candidate for the trans-abdominal approach.

## Introduction

An intrapericardial diaphragmatic hernia (IPDH) is the rarest type of non-diaphragmatic hernia in adults [1]. In an IPDH, abdominal viscera such as the small bowel herniates through the diaphragm up into the chest cavity penetrating the pericardium [1]. This is highly unusual because the pressure from the heart often offers enough superior support to the left side of the diaphragm to prevent herniation [2]. Most often, IPDH is a sequela of some form of blunt trauma to the abdomen or chest including severe blows to the chest, deceleration trauma while traveling in a vehicle, and falls in the elderly [2]. Clinical manifestations of IPDH vary from patient to patient depending on the type of abdominal tissue and the amount of abdominal contents that herniate through the diaphragm into the chest cavity. Common presenting symptoms in patients with IPDH are nonspecific chest or abdominal pain, shortness of breath, vomiting, and gastrointestinal discomfort [2]. On physical examination, bowel sounds may be heard in chest fields, indicating a herniation of the bowel through the diaphragm. Decreased breath sounds or distant heart sounds may be heard as well [2]. This case report describes an IPDH following a 10 cm diaphragmatic rupture in an 80-year-old woman presenting after being involved in a motor vehicle collision (MVC). This patient presented with bowel sounds in the chest, and largely no additional complaints. A post-traumatic diaphragmatic rupture was suspected and the patient was taken to surgery for repair of the diaphragmatic defect. During laparoscopic repair, it was discovered that the patient had an IPDH.

## Case presentation

**Figure 1:**
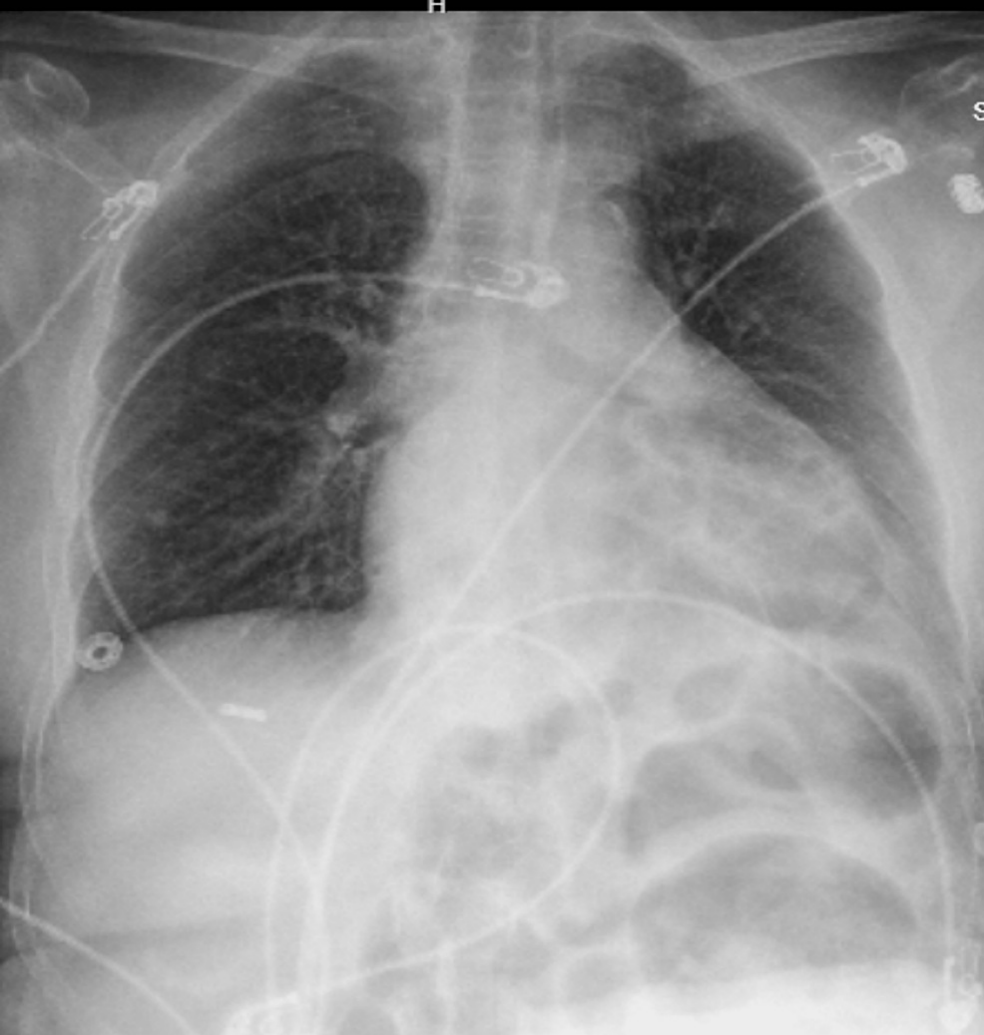
Chest x-ray depicting bowel in the thoracic cavity

An 80-year-old female with a past medical history of hypertension (HTN) and seasonal allergies and no significant past surgical history presented to Carle Foundation Hospital on 10/17/2022 after a MVC. The patient was leaving church when she was suddenly struck on the front driver’s side by a vehicle traveling at an approximate speed of 60 mph. The primary survey was significant for systolic blood pressure of 66 mmHg so she received 2 liters of crystalloids and a unit of blood to which she responded appropriately, and her blood pressure normalized. The secondary survey demonstrated small abrasions to the left dorsal hand and the right pretibial area. On presentation, the patient had a hemoglobin (Hgb) of 8.8 and otherwise unremarkable laboratory values. In addition, an electrocardiogram (EKG) was performed and it demonstrated a right bundle branch block (RBBB) without ST elevations or depressions. Chest x-ray (CXR), pelvic x-ray, and a computed tomography angiography (CTA) of the chest, abdomen, and pelvis demonstrated findings significant for an IPDH with small bowel emanating through the thoracic cavity and into the mediastinum (Figures 1 through 3).

**Figure 2:**
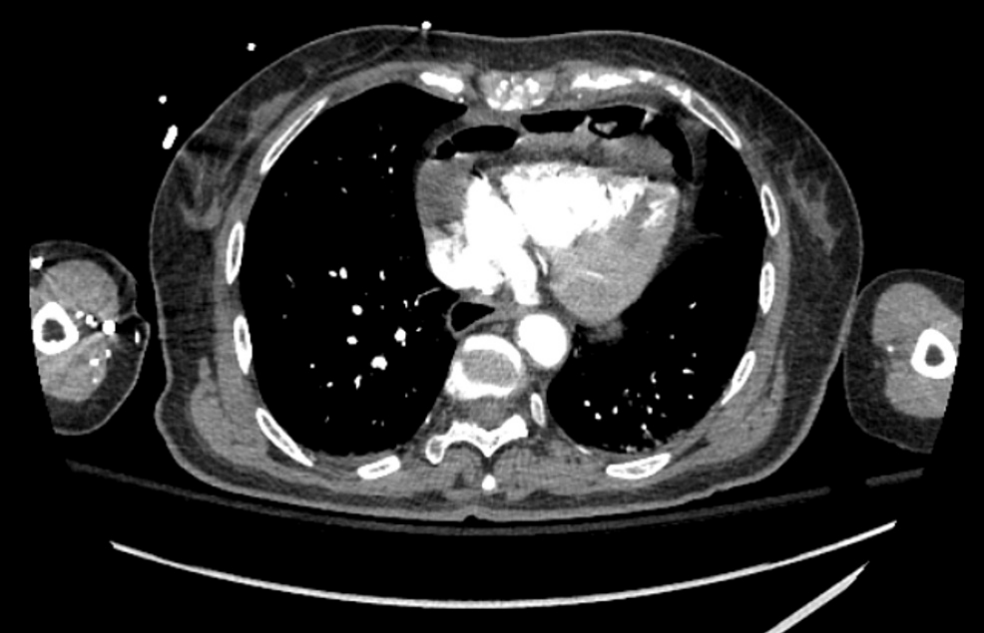
Computed tomography angiography of the chest revealing bowel penetration into the mediastinum (axial view)

**Figure 3:**
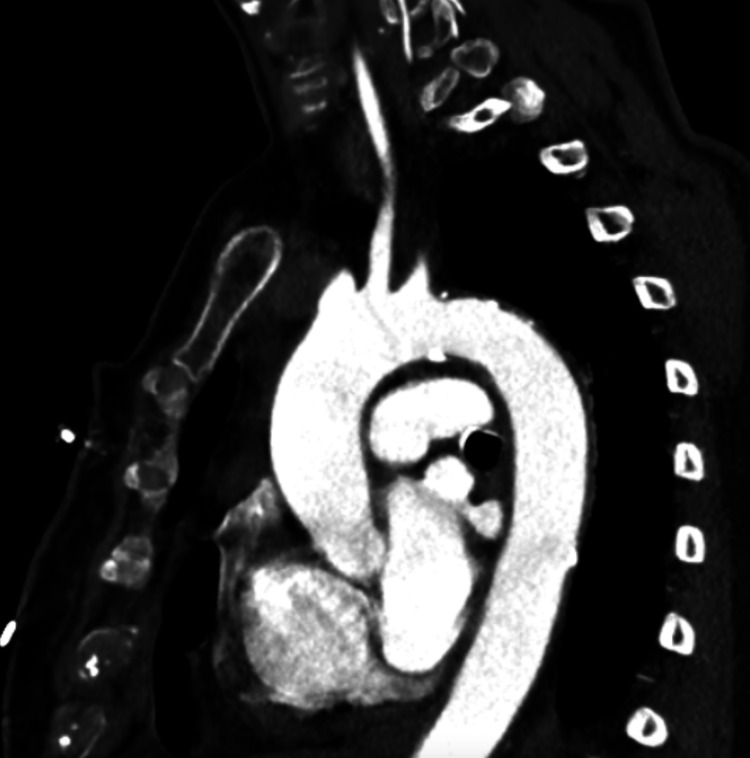
Computed tomography angiography of the chest revealing bowel penetration into the mediastinum (sagittal view)

**Figure 4:**
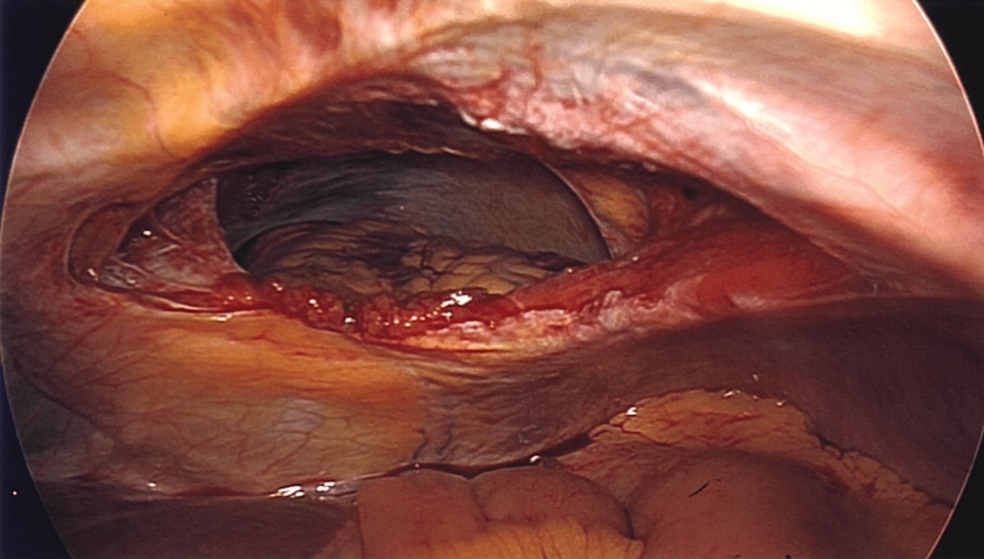
Direct visualization of the heart via 10-cm diaphragmatic defect and pericardial sac defect

**Figure 5:**
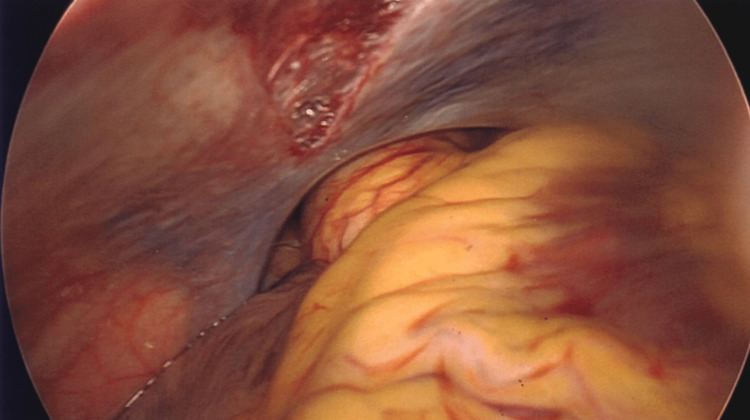
Direct visualization of the heart and ascending aorta via 10-cm diaphragmatic defect and pericardial sac defect

**Figure 6:**
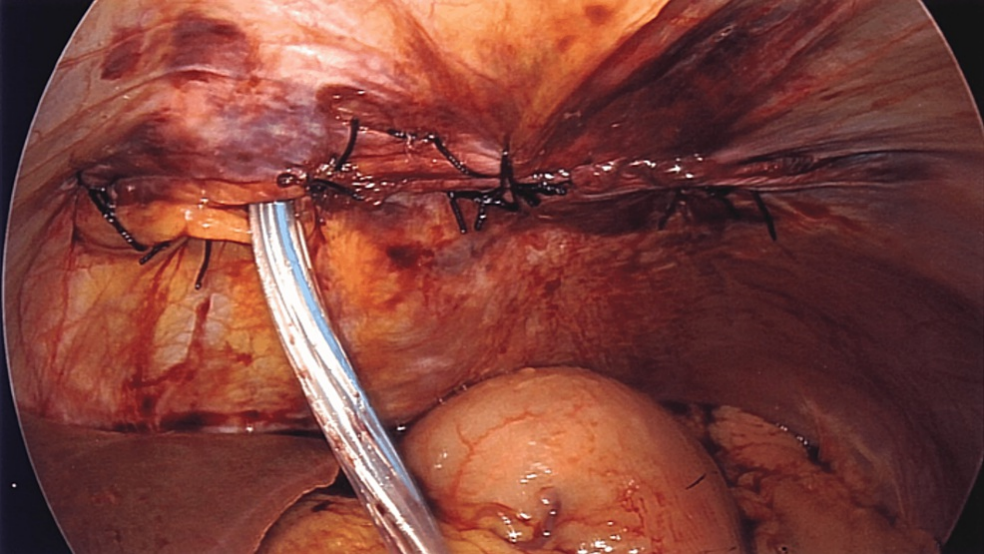
Diaphragmatic defect repair with drain placement

After the patient was evaluated in the emergency department (ED) by the trauma team, she was taken to the operating room for laparoscopic primary repair of traumatic IPDH with the placement of a 19 French pericardial drain. After gaining intra-abdominal access via the open Hasson technique, an inspection of the diaphragm revealed a large 10-cm defect that allowed for direct visualization of the heart as there appeared to be a defect within the pericardial sac as well (Figures 4 and 5). Loops of bowel were not visualized within the defect, likely due to reduction at the time of induction or perhaps when in the ED as the patient had been placed in reverse Trendelenburg in preparation for the operating room (OR). A 19 French Blake drain was placed over the heart, into the abdomen, and emerging extracorporeally through an abdominal incision. Multiple 2-0 silk sutures were used in a horizontal mattress fashion to close the defect primarily around the drain (Figure 6). On the first postoperative day (POD#1), an echocardiogram was performed and her cardiac function was noted to be normal. She was seen by our orthopedic surgery team for her pelvic fractures which were all managed conservatively. Her postoperative course was unremarkable and on POD#3, the pericardial drain was removed. The patient was subsequently discharged on POD#4.

She was seen in the clinic 2 weeks postoperatively and was deemed to be doing well. At the time of this publication, the patient has resumed her baseline activities and is devoid of any symptomatic sequelae.

## Discussion

This case illustrates a rare complication of blunt trauma sustained by a patient involved in an MVC: an IPDH. Post-traumatic IPDH is an extremely infrequent complication of blunt force to the chest or abdomen. According to a literature review published by Reina et al. in 2001, at that time only approximately one post-traumatic IPDH was reported yearly [3]. Additionally, diaphragmatic rupture, most often caused by trauma, accounts for less than 1% of all cases of IPDH, making this case even more unique. Other causes of IPDH include defects in the central tendon of the diaphragm that can be congenital or trauma-related [4]. IPDH may happen during trauma, with MVCs being the most common trauma-induced cause of this complication when a sudden increase in intra-abdominal pressure occurs secondary to a sudden change in direction of travel (i.e. deceleration injury). Notably, it can also occur when the external application of force to the anterior trunk occurs. In this patient’s case, she was T-boned during the MVC. Therefore, it is likely that her IPDH was caused by a sudden change in direction of movement leading to increased intra-abdominal pressure causing herniation of abdominal contents through the left side of the patient’s diaphragm up into the pericardium leading to its rupture. Although the preoperative images were suggestive of small bowel herniation, the transverse colon, the stomach, and the greater omentum are generally the most common abdominal organs to herniate through the diaphragm and pericardium in an IPDH [4].

The treatment for an IPDH is surgery in the acute and in the delayed setting. In acute traumatic IPDH, a trans-abdominal approach is preferred because it allows for better visualization of the diaphragmatic defect that needs to be repaired. In cases of delayed IPDH, a thoracic approach is used due to the increased likelihood of pericardial adhesions that need to be addressed [4]. Using the trans-abdominal approach, this patient’s diaphragmatic tear was able to be sutured closed laparoscopically without complication.

A common and feared complication of IPDH, especially in a delayed setting, is cardiac tamponade [5]. Cardiac tamponade is the accumulation of fluid in the pericardium leading to compression of the heart. Without prompt treatment, this fluid accumulation will cause pressure to build up around the heart not allowing it to fill properly and leading to reduced cardiac output. This can be devastating and fatal. In addition to cardiac tamponade, the delayed diagnosis of IPDH has a higher likelihood of developing pericarditis or other infectious processes in the thoracic cavity due to contact with abdominal contents. Notably, if concomitant intestinal rupture occurs, the sequelae can be quite morbid. These complications punctuate how important it is that the diagnosis of IPDH is done promptly. Since presenting symptoms are typically vague, a careful physical exam should be performed and imaging such as a CXR and/or thoracic CT should be done to avoid misdiagnosis.

## Conclusions

While rare, IPDH should be promptly diagnosed given the increased morbidity associated with a misdiagnosis. Since the presentation can have an elusive constellation of symptoms, it can easily be overlooked on initial history and physical exam. However, if imaging confirms the diagnosis, prompt surgical intervention should be undertaken. In the acute setting, a trans-abdominal approach is preferred as it provides the ability to perform a diagnostic evaluation of intra-abdominal contents for any concomitant injury and it provides a better visualization of the diaphragmatic defect. In our case, a laparoscopic repair proved to be a viable option with quick recovery - an important consideration, especially in the elderly patient population.
